# COVID-19 real-world data for the US and lessons to reopen business

**DOI:** 10.1371/journal.ppat.1008756

**Published:** 2020-08-27

**Authors:** Pascal J. Goldschmidt-Clermont

**Affiliations:** Lennar Corporation, Miami, Florida, United States of America; Vallabhbhai Patel Chest Institute, INDIA

## Abstract

This Pearl article recounts the story of a US corporation, Lennar, the nation’s leading homebuilder, an essential function in the US (not allowed to lock down), when faced with the coronavirus disease 2019 (COVID-19) pandemic at the end of February 2020. The culture of the company, which allowed it to proceed safely, is one of cohesion, trust, teamwork, and respect for fellow humans. Theirs is a culture in which the safety, wellness, and health of the associates (employees) and the communities they serve is the number one priority. All associates wear a name badge with first name only, and all name badges share the same family name, Lennar. At Lennar, individual success means nothing, and collective success means everything. This is the story of how Lennar took control of the COVID-19 pandemic, metamorphosed itself into an even stronger organization, better suited to deal with COVID-19, and more importantly, optimally suited for the 21st century. The lessons learned not only were instrumental to Lennar but could also apply to any company eager to reopen their business.

## Overview

In March of 2020, the coronavirus disease 2019 (COVID-19) pandemic had expanded to the United States. Companies designated as “essential” for the US had to maintain productivity in spite of the growing threat created by the severe acute respiratory syndrome coronavirus-2 (SARS-CoV-2) virus. With this report, we present the response of one such company, the Lennar Corporation, the nation’s leading homebuilder in the US. Within days, Lennar had implemented a morning health check via its enterprise resource planning (ERP) system, to identify associates (employees) who were sick, or not in their “usual state of health.” With this survey, Lennar was able to ensure that sick associates would not show up to work and, instead, would self-quarantine at home. Furthermore, with thorough contact tracing, associates exposed to COVID-19 patients (suspected or reverse transcription [RT]-PCR-test–confirmed) were also asked to self-quarantine. Through this survey—in addition to other safety measures, such as an overhaul of the company with nearly 50% of the company working from home in response to the virus, prolific communication, and many more measures—Lennar was able to function safely for its associates and successfully as an enterprise. The data that we present here are “real world data” collected in the context of working throughout a dreadful pandemic, and the lessons learned could be helpful to other companies that are preparing to return to work.

## COVID-19

SARS-CoV-2 became a human health threat during the second half of 2019 starting in the city of Wuhan in China and, by the first quarter of 2020, led to a global pandemic [1]. SARS-CoV-2 can cause acute respiratory distress syndrome (ARDS) and seems to be more dangerous and contagious than SARS-CoV-1 [2]. Until recently, our information on the prevalence of the pandemic was limited to data collected on people who were tested and were found positive for coronavirus disease 2019 (COVID-19; 2,215,618 total cases on June 19, 2020) and—among those positive individuals—the fraction of people who died (approximately 5% of total) [3,4].

While helpful in justifying the “lockdown order,” a limitation of such data is that they are impacted markedly by guidelines (local and national) about who could be tested [5], and in the US, our real-world experience is that the majority of individuals who have presented with typical COVID-19 symptoms could not get tested because of limited supply of test kits. Indeed, Bendavid and colleagues have shown that by early April, seroprevalence of antibodies specific for CoV-2 for a cohort of volunteers recruited in Santa Clara County, California, was much greater than the number of confirmed cases reported for the same region [6]. Furthermore, because the bias for testing appears to lean toward sicker patients, this also affects the reported death rate [5,6].

In spite of the severity of the pandemic, some businesses were asked to maintain productivity during the “lockdown period” because they are providing an essential function in the US. Lennar Corporation is such a business (Lennar, New York Stock Exchange [NYSE]: LEN and LEN.B). Lennar builds approximately 50,000 homes yearly, present in 21 states and ≥78 markets from coast to coast, including areas affected by COVID-19 hot spots. Lennar employs about 10,000 associates (employees), a cohort that matches the race and social US distribution. The age range for the company is 18–83 (median age of 49). The response of this company to the COVID-19 challenge informs an important knowledge gap relative to industry and the COVID-19 pandemic and might be of interest particularly as businesses are in the process of reopening.

## Strategy

**Fig 1:**
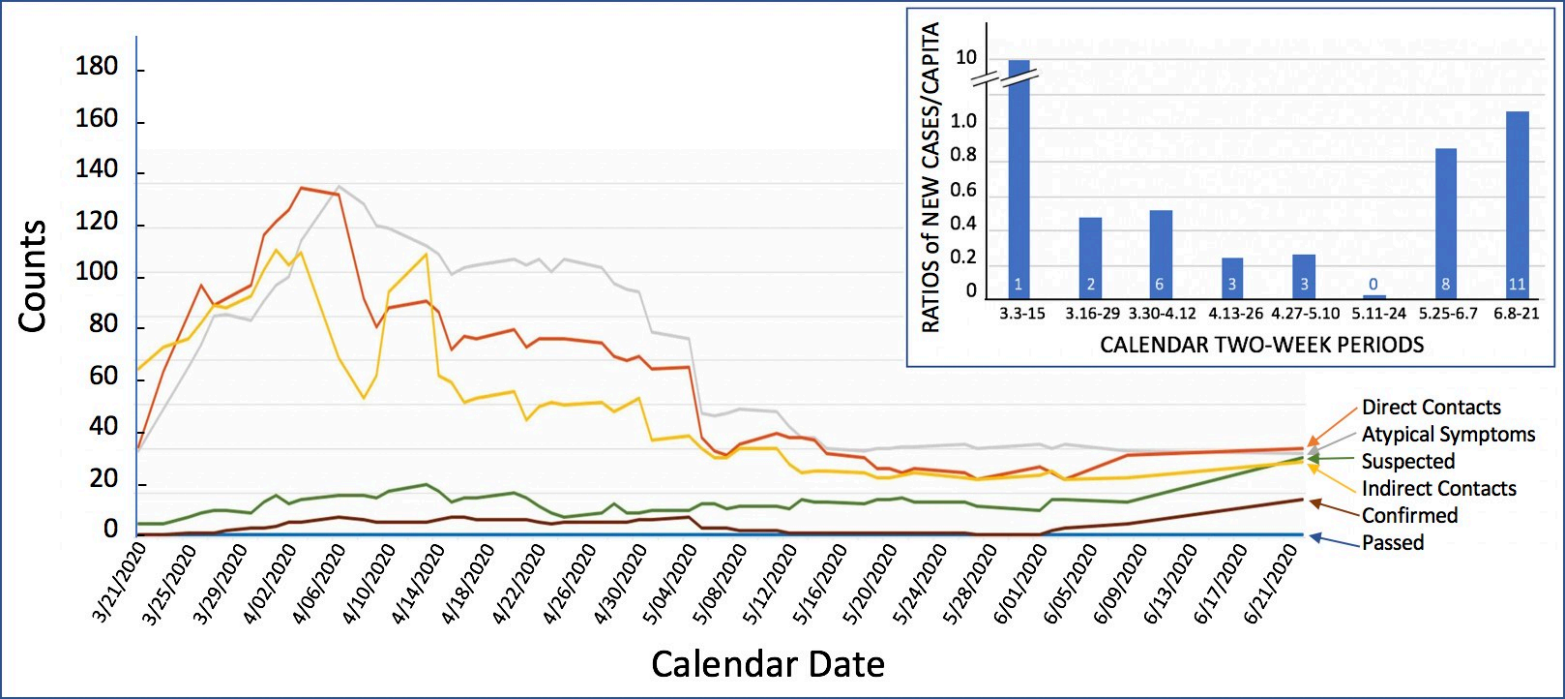
Line graph recounting the key categories of risk groups: Passed (deceased), Confirmed (test-confirmed cases of COVID-19), Suspected (cases of COVID-19), Direct Contact (with COVID-19 patient, suspected or confirmed), Indirect Contact (contact with direct contact, etc.), Atypical Symptoms (cases with symptoms of non-COVID-19 communicable illness), and how they evolved over time. This trends database, which represents the balance between new quarantine occurrences and return to work events at end of quarantine, was started on March 21, not 19. Note that most risk categories peaked initially between March 30 and April 15, and then decreased thereafter until Memorial Day Weekend, 2020, when the numbers of suspected and confirmed cases started to increase again. The total number of COVID-19 test-confirmed associates was 34 from March 3 till June 21, 2020. Inset: Ratios are calculated by adding all new cases for each 2-week period (from March 3 to June 21, 2020) at Lennar, divided by 10,000 (total number of employees in the company), and as denominator, new cases in the US reported during the same 2-week periods divided by 330,000,000 (population of the US). Numbers at the bottom of each column represent the new cases at Lennar during each 2-week period. COVID-19, coronavirus disease 2019.

At the beginning of March, Lennar started collecting real-world data, the Daily Health Check (DHC), via a survey powered by information generated by the Centers for Disease Control and Prevention (CDC) guidelines, to ensure the safety of its associates. All 10,000 associates were asked at the beginning of the day, before leaving for work, questions about their health specific for COVID-19 (see Table 1 of reference [7] for details) and based on ethical guidelines provided by the Equal Employment Opportunity Commission (EEOC). The DHC survey, which was conducted using the company’s ERP system (Workday), started on March 19, 2020, and continued till present. This database was further refined for people whose response indicated that they were sick, had contact with someone who was sick, or had traveled, using Lennar’s information extracted from its ERP system (see Fig 1 of reference [7] for details), by asking additional questions about contacts, symptoms, or travels via ERP and phone calls. Efforts were made to ensure that associates with no response and those not contacted were not particularly sick or incapacitated.

In addition to this giant survey, many technologies were recruited to ensure the safety, health, and wellness of the associates. All associates with symptoms were encouraged to ask their care provider to be tested for COVID-19. Testing for COVID-19 was done locally, where the associates live, and 100% of the testing was done using nasopharyngeal swab and Nucleic Acid Amplification for SARS-CoV-2 (NAA testing). All associates with symptoms of upper-airway communicable illnesses were quarantined. Quarantine was 14 days or more (due to the requirement to be symptom free for 3 days without use of symptom-suppressing drugs) for those suspected of having COVID-19 (one or more typical COVID-19 symptoms according to the CDC and/or diagnosed as CDC persons under investigation [PUIs] by their personal care provider). Some PUIs were confirmed to have COVID-19 with a positive COVID-19 test. However, our real-world experience is that the majority of individuals who have presented with typical COVID-19 symptoms could not get tested until May 2020 because of limited test kit supply in the US. Quarantine was 7 days for those suspected of having other upper-airway communicable disorders, based on their atypical symptoms (runny nose, sneezing, conjunctivitis, etc.). Contact tracing was conducted with the completion of a thorough list of contacts for anyone suspected (PUI) or known to be infected (positive COVID-19 test), with review of all associates, customers, trade partners, family, friends, and/or children they may have been in contact with. The contact tracing list was then used to request self-quarantine for any associate with direct (14 days or longer if they became symptomatic) or indirect (7 days) contact with a COVID-19-suspected or test-confirmed individual (see Table 1 legend of reference [7], for further details on direct and indirect contacts).

Physical distancing was also supported by other interventions, such as 50% of the associates working from home (working from home was not an option prior to our response to COVID-19) and using physical distancing (6 feet minimum between associates) for associates at work facilities. The availability of personal protection equipment (PPE) was implemented, with wearing of a surgical face mask or face cloth in public, and latex gloves as needed. Hand washing or sanitizing was made available at any work site, including on the field. Thorough cleaning of all facilities and new homes was performed regularly with US Environmental Protection Agency (EPA)-certified products able to destroy CoV-2, as well as daily scrubbing of surfaces also with EPA-approved COVID-19 disinfectants. Additional measures included the following: discontinuation of all in-person meetings, a quarantine of 14 days for all associates after international travels or cruises and of 4 days once back home from domestic travels (or more if they were to develop symptoms), and a massive information campaign to inform associates of the latest news on the pandemic and the company response (via multimedia: company video, weekly publications, direct emails for key messages, posters at all work sites, human resource calls, Microsoft Teams, Zoom, and other meeting systems, consults on complex issues, etc.).

## Real world data

Based on this cohort of approximately 10,000, which we continue to survey daily, Lennar was able to generate a database populated by >5,000,000 individual data points since March 19, 2020. The response rate to the DHC survey was constant at approximately 90%. In total, from March 19 to June 3, 2020, 18 associates were quarantined for COVID-19 test confirmed infection. This group peaked between March 31 and May 4, then was near zero, before it peaked again after Memorial Day, 2020 (Fig 1). Other associates were quarantined because they were suspected of having COVID-19 (PUI), either because they had typical symptoms or because their care providers had diagnosed them with COVID-19. Some of these individuals were able to get tested depending on the availability of COVID-19 tests in their geographical area. This group peaked around April 13 and then plateaued (Fig 1). Another group of associates was quarantined because of symptoms of other upper respiratory tract infections, symptoms typically not associated with COVID-19, and including sneezing/rhinorrhea, conjunctivitis, etc. This group peaked on April 6, and then progressively decreased (Fig 1), and there was limited opportunity to test this group at all for COVID-19 until May 2020, in spite of our encouragement to get tested.

Looking further at trends, direct contacts peaked on April 3, and indirect contacts peaked on April 1 and then progressively dropped (Fig 1), suggesting either that there were fewer COVID-19 individuals to have contact with after April 3 or that associates were less aware of their contacts (or yet other explanations). The test-positivity rate of the COVID-19 testing did also drop, from a near 30% at the end of March to 0% for a couple of weeks in May 2020, in spite of the increased availability of testing. Then, starting on May 25, we saw a recrudescence of positive COVID-19 tests and an increase in test-positivity rate, especially on Memorial Day weekend. The drop of test-positivity rate in May was likely explained in part by an increased availability of testing for COVID-19 [8–10], but it was also due to fewer new cases within the company and until the recent uptick. Indeed, when new COVID-19 cases are expressed as ratios between the count of new cases for Lennar divided by total Lennar associates (10,000) and—as denominator—new US cases divided by total US population (330,000,000), for each of the first eight 2-week periods, this ratio decreased rather markedly over time (Fig 1, inset). The improvement of Lennar COVID-19 new cases relative to that of the US may have resulted, at least in part, from our efforts to prevent new COVID-19 infections. The sudden return of new cases at Lennar in June 2020 is of concern and has involved both associates working from home and those at worksites (approximately 50/50), an uptick that was detected in our database before it became detectable on the CDC national count of new cases [3]. It is now clear that there is a recrudescence of new cases and test-positivity rate in the US, and it is once again a challenge to get tested, especially in states that are experiencing the greatest increases in new cases.

## Conclusion and limitations: Lessons learned, and how to reopen US industry safely

SARS-CoV-2 has 2 dreadful impacts: first, the severe health consequences and, second, an economic downturn. Our database became invaluable in helping the company make essential interventions. The most important of all was to make sure, using our DHC survey, that people who were sick, or simply not “in their usual state of health,” would never come to work and instead would self-quarantine. It turns out, retrospectively, that even if they were to come to work at all and only for a few minutes, it would be enough time—while in a queue or while getting a temperature check (temperature should be checked at home before going to work)—to increase the risk of contamination of others. The process of self-quarantining for any associate that would represent a contagious risk for others, by making sure that they would not come to any work facility, was our most effective intervention (Box 1). However, even at the peak of this pandemic, less than 5% of our associates were quarantined, thus allowing the company to remain fully functional. It has been shown that even asymptomatic COVID-19-infected individuals can infect others [11]. Hence, when tracing contacts for COVID-19 patients, we also searched 48 hours prior to them becoming symptomatic, to make sure we would catch all contacts during this pre-symptomatic but contagious period. According to our data, we were able to eliminate contamination of associates at the workplace, due to contact tracing and immediate quarantining of symptomatic COVID-19 patients and their (mostly) asymptomatic contacts. Without feedback from real-world data, it would be difficult for any organization to find out whether guidelines are being followed and successful in preventing COVID-19 infections. Hence, getting data is critical to effective population health management throughout this COVID-19 pandemic and, hopefully, will at some point be further helped by the availability of successful drug(s) and/or vaccine(s).

**Box 1. Preventive measures ranked for effectiveness to prevent COVID-19 outbreaks**

1. Getting data. Digital DHC in the morning from home provides unique information and data that are critical to managing and reducing the impact of CoV-2, by preventing infected employees from arriving at a work place and contaminating others, instead asking them to self-quarantine at home. The DHC is also essential to tracing contacts.
2. Limiting and tracing contacts. Allowing those whose presence at a workplace is not essential to work from home reduces crowding of work sites and social contacts. Likewise, tracing contacts of infected individuals and asking them to get tested and self-quarantine at home while waiting for results limits infections.
3. Testing. Knowing who is infected and who is not is important for measuring the impact of guidelines over time and for dealing with hot spots for COVID-19. It can also accelerate return to work for those who are quarantined (Contacts especially). Access to antibody testing has remained too limited for us to assess its role in the real world.
4. Distancing (6 feet/2 meters). Keeping at a distance from others at work sites and other public places limits transmission of CoV-2. Following the evolution of COVID-19 hot spots across the US and staying away from them also helped reduce infections. Eliminate in-person meetings.
5. Promoting strong hygiene. Frequent hand washing (20 seconds or more) and sanitizing is critical to reduce access of CoV-2 to entry sites for the body: mouth, nose, and eyes. Scrubbing surfaces thoroughly with EPA-certified products capable of killing CoV-2 helps sanitizing work sites, especially if within a hot spot.
6. Using personal PPE. The effectiveness of PPE is strongly dependent on the quality of the equipment, and PPE such as surgical face masks are efficient at protecting others—and possibly the person wearing the face mask—from infection.
7. Informing. A clear and transparent multimedia campaign to keep employees informed and engaged contributes to effective prevention and adherence to guidelines.

Companies that want to return to productivity—from large corporations to small businesses—could indeed keep the proliferation of their COVID-19 cases under control. However, it is important to also stress the limitations to our real-world observation: (a) we had no control of who among our associates could get tested for COVID-19; (b) our guidelines were focused on associates becoming symptomatic, while a large fraction of COVID-19 patients are not displaying symptoms, and our success in limiting transmission of COVID-19 at work sites suggests that the rate of infection by truly asymptomatic carriers (not pre-symptomatic COVID-19 patients) is rather low [12]; and (c) the success of our approach was strongly dependent on the culture of the organization, where teamwork, trust, and respect for fellow humans are key values. It is uncertain whether organizations without such values would be able to produce comparable results. Nevertheless, we believe that strategies pursued by Lennar since the beginning of March 2020—if generalized to companies eager to reopen—could minimize viral transmission and maximize safety, health, and wellness within their workforce and the communities they serve [13,14], while maintaining productivity.

## References

[1] Zhu N, Zhang D, Wang W, Li X, Yang B, Song J, et al (2020) A novel coronavirus from patients with pneumonia in China. N Engl J Med 382: 727–733. 10.1056/NEJMoa2001017 31978945 PMC7092803

[2] Fauci A, Lane H, Redfield R (2020) Covid-19—Navigating the Uncharted. N Engl J Med 382: 1268–1269. 10.1056/NEJMe2002387 32109011 PMC7121221

[3] CDC (2020) Coronavirus disease 2019 (COVID-19), cases, data and surveillance. [cited 2020 June 21]. https://www.cdc.gov/coronavirus/2019-ncov/cases-updates/cases-in-us.html.

[4] Johns Hopkins Center for Systems Science and Engineering (2020). Coronavirus COVID-19 Global Cases. [cited 2020 June 21]. https://coronavirus.jhu.edu/map.html.

[5] Lipsitch M, Swerdlow D, Finelli L (2020) Defining the Epidemiology of Covid-19—Studies Needed. N Engl J Med 382: 1194–1196. 10.1056/NEJMp2002125 32074416

[6] Bendavid E, Mulaney B, Sood N, Shah S, Ling E, Bromley-Dulfano R, et al. (2020) COVID-19 Antibody Seroprevalence in Santa Clara County, California. medRxiv. Preprint. [cited 2020 June 21]. https://www.medrxiv.org/content/10.1101/2020.04.14.20062463v2. 10.1093/ije/dyab010 PMC7928865 33615345

[7] Goldschmidt-Clermont P (2020) COVID-19 Real-World data for the US and lessons to re-open business. medRxiv. Preprint. [cited 2020 June 21]. https://www.medrxiv.org/content/10.1101/2020.06.02.20120618v1.

[8] Meyer R, Madrigal A (2020) A New Statistic Reveals Why America’s COVID-19 Numbers Are Flat. The Atlantic. [cited 2020 June 21]. https://www.theatlantic.com/technology/archive/2020/04/us-coronavirus-outbreak-out-control-test-positivity-rate/610132/.

[9] CDC (2020) Testing in the US. [cited 2020 June 21]. https://www.cdc.gov/coronavirus/2019-ncov/cases-updates/testing-in-us.html.

[10] Sanger-Katz M, Richtel M (2020) Despite promises, testing delays leave Americans “flying blind”. The New York Times. [cited 2020 June 21]. https://www.nytimes.com/2020/04/06/health/coronavirus-testing-us.html.

[11] Gandhi M, Yokoe D, Havlir D (2020) Asymptomatic transmission, the Achilles’heel of current strateguies to control COVID-19. N Engl J Med 10.1056/NEJMe2009758 32329972 PMC7200054

[12] Van Kerkhove M (2020) WHO health Emergencies Programme. [cited 2020 June 21]. https://www.forbes.com/sites/mattperez/2020/06/08/who-says-asymptomatic-spread-of-coronavirus-very-rare-but-experts-raise-questions/#72de0f0443d0.

[13] Flaxman S, Mishra S, Gandy, Unwin J, Mellan T, Coupland H, et al (2020) Estimating the effects of non-pharmaceutical interventions on COVID-19 in Europe. Nature 10.1038/s41586-020-2405-7. 32512579

[14] Hsiang S, Allen D, Annan-Phan S, Bell K, Bolliger I, Chong T, et al (2020) The effect of large-scale anti-contagion policies on the COVID-19 pandemic. Nature 10.1038/s41586-020-2404-8. 32512578

